# Room-temperature mL-to-μL quantitative liquid concentration device for cyclone flow

**DOI:** 10.1007/s44211-024-00654-z

**Published:** 2024-08-30

**Authors:** Hidekatsu Tazawa, Kazuma Mawatari

**Affiliations:** Graduate School of Information, Production and Systems, Waseda University, 2-7 Hibikino, Wakamatsu, Kitakyushu, Fukuoka 808-0135 Japan

**Keywords:** Liquid concentration, Cyclone flow, Quantitative analysis, Thermal lens detector, Environmental analysis, Microfluidic devices

## Abstract

Highly sensitive quantitative analysis of liquids is required in various fields. Analytical instruments and devices such as chromatography, spectroscopic analysis, DNA sequencers, immunoassay, mass spectrometry, and microfluidic devices are utilized for this purpose. Typically, the sample volume is at the milliliter scale, while the analysis volume is at the microliter scale. Consequently, most of the sample is discarded. Therefore, a universal volume interface is required to quantitatively concentrate samples from milliliter to microliter volume. This study introduces a liquid quantitative function to the cyclone concentration method using a millimeter-scale channel, which is highly suitable for controlling liquids at the microliter scale due to its high fluidic resistance against cyclone flow. This method enables the effective control of liquid concentration by cyclone flow. The optimum channel structure is investigated, and a 33-fold concentration of aqueous solutions is demonstrated. Finally, the concentration device is applied to measure molybdenum ions in a river.

**Graphical abstract:**

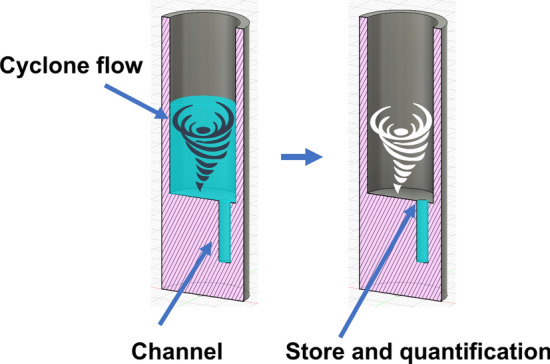

**Supplementary Information:**

The online version contains supplementary material available at 10.1007/s44211-024-00654-z.

## Introduction

Liquid analysis is widely used in various fields, such as chemistry, biotechnology, food analysis, and environmental analysis [1–3]. In particular, concentration determination methods [4] are widely used in various analytical methods, including separation analysis such as liquid chromatography [5, 6], mass spectrometry [7], spectroscopic analysis such as light absorptiometry [8], and fluorescence spectrometry [9, 10]. Immunological analysis (e.g., enzyme-linked immune-sorbent assay) [11] and nuclear magnetic resonance [12] also rely on accurate concentration determination. Microfluidic devices are also used for high-performance analysis [13, 14], facilitating reactions and measurements in narrow channels that typically range from tens to hundreds of millimeters in diameter. These microfluidic platforms offer advantages such as a small sample size, high-speed reactions, and compact size.

Various approaches have been explored for the highly sensitive analysis of low-concentration liquid samples. One strategy involves improving the detection method [15]. For instance, transitioning from fluorescence detection to chemiluminescence detection (chemiluminescent labeling) is used in medical diagnosis [16]. However, enhancing the detection method can be challenging, necessitating more general approaches. Another approach involves concentrating the liquid sample before measurement. Typically, the available sample volume is at the milliliter scale, whereas the actual volumes utilized in analytical instruments and microfluidic devices are at the microliter and nanoliter scales, respectively. Consequently, the analytical instrument only considers a minimal volume of the sample, and most of it is wasted. If the liquid volume can be quantitatively converted from the milliliter to the microliter scale at room temperature, most analytical methods, including those involving biological samples, can achieve highly sensitive analysis without altering the detection method.

Several methods are available to concentrate liquid samples: rotary evaporators [17, 18], nitrogen spraying methods [19], and ultrafiltration methods [20]. However, quantitative concentration is currently difficult at the milliliter scale. Rotary evaporators face challenges in observing liquid volume due to their rapid rotary motion. Nitrogen spaying can cause liquid scattering due to gas pressure. Both methods require several hours for concentration, and their applications are limited to biological samples due to heating acceleration.

**Fig. 1:**
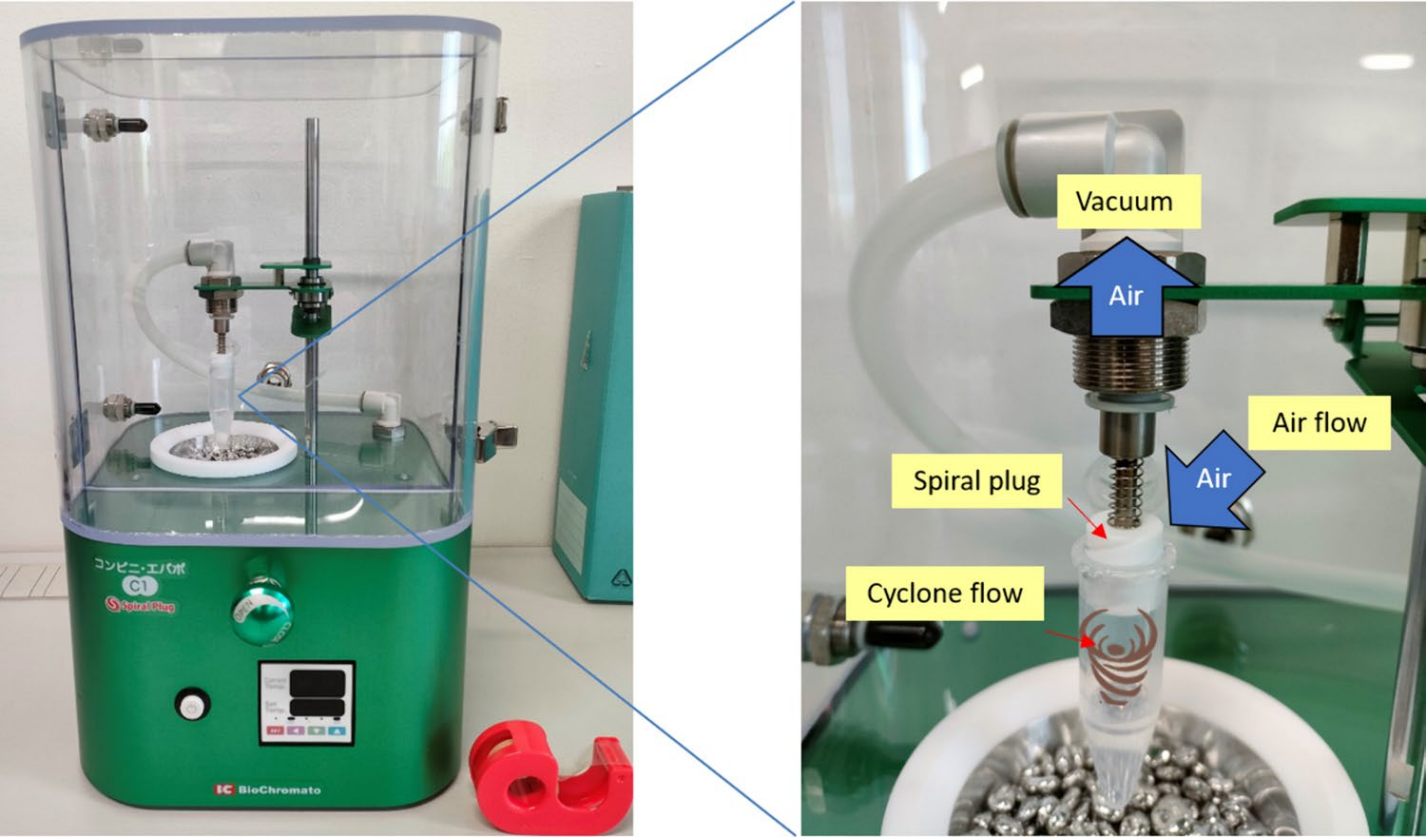
Cyclone concentration method and equipment

While ultrafiltration is an effective method, it cannot guarantee the collection of all analyte molecules, and liquid may remain inside the membrane, limiting its application to large molecules. A cyclone-based concentration method [21, 22] is used to concentrate small volumes of liquid at room temperature, as shown in Fig. 1. This method utilizes negative pressure and a spiral plug (a plug with a helically grooved channel) to create cyclone flow inside a tube. Cyclone flow increases the air/liquid interface, allowing the concentration of a 1-mL liquid sample at room temperature for 30 min.

However, controlling the liquid volume remains challenging due to the turbulent flow induced by the cyclone. Therefore, the method is primarily used to dry the liquid completely. There is a critical need for improved liquid control methods.

In this study, we proposed a method to control the liquid volume at the milliliter scale using the cyclone-based concentration method. A millimeter-scale channel is created in a concentration tube using a 3D printer. The millimeter-scale channel is expected to halt cyclone flow when it reaches the channel entrance due to its higher fluidic resistance than a centimeter-scale tube. We investigate the optimal channel structure, demonstrate quantitative concentration, and apply this to an environmental analysis of molybdenum ions in river water.

## Materials and methods

### Design and fabrication of the concentration device

We utilized a cyclone concentration system (Smart Evaporator C1, BioChromato Co., Ltd. Japan) to perform the quantitative concentration. This system employed a Polytetrafluoroethylene plug with spiral channels placed in a microtube containing the sample solution. Subsequently, the air was aspirated by a vacuum pump. Due to the low pressure in the microtube, the air outside the microtube entered through the spiral plug’s channels, generating a cyclone airflow inside the microtube. The cyclone airflow induced the liquid cyclone flow due to shear stress at the air/liquid interfaces. Owing to the enhanced gas–liquid contact area, the liquid was efficiently concentrated at room temperature.

**Fig. 2:**
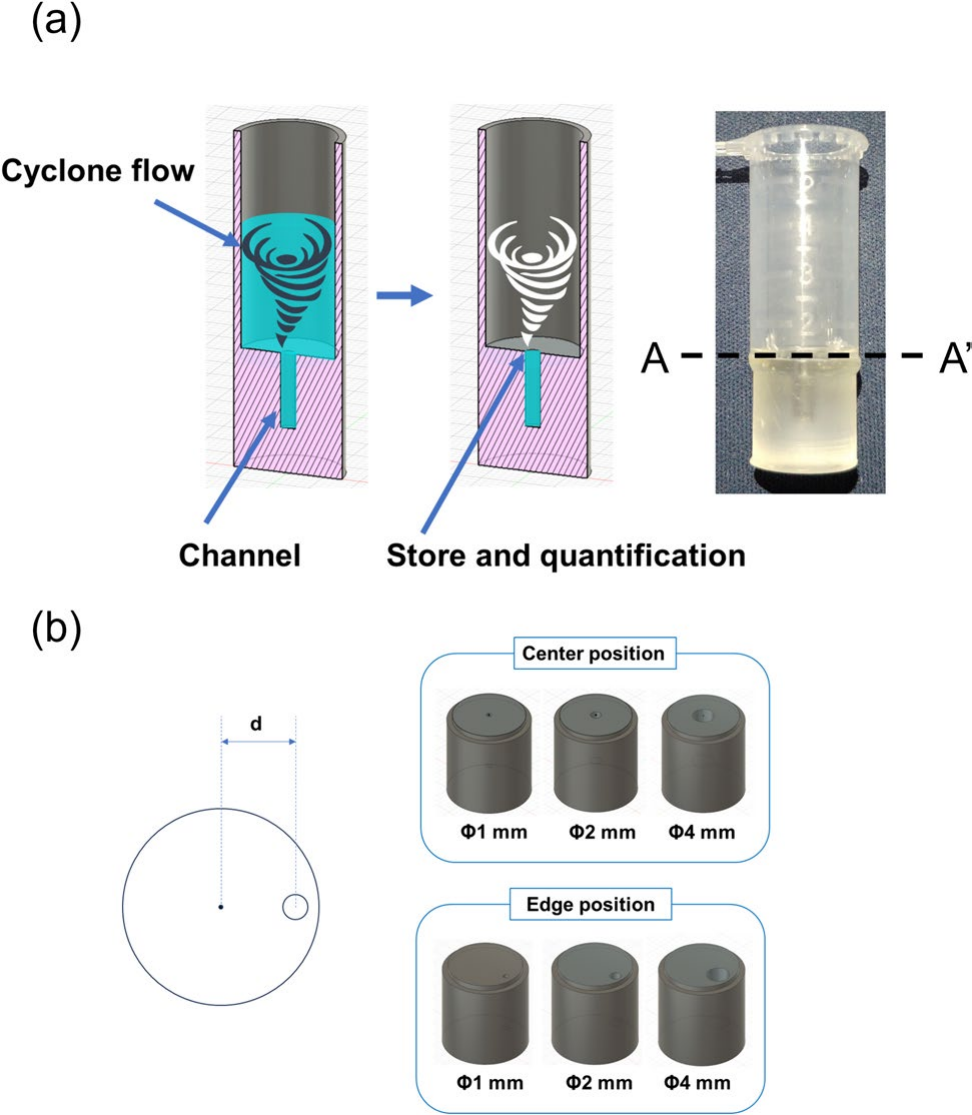
**a** Cyclone-based quantitative concentration method and device. **b** A–A’ cross-sectional view and position of the channel

To determine the concentration quantitatively, millimeter channels were fabricated at the bottom of the concentration device, which inhibited cyclone flow and significantly delayed the drying rate. Consequently, the millimeter-scale channel could function as a milliliter-scale pipet, as shown in Fig. 2a. A 5 mL microtube (EPPENDORF. AG) was cut, and the concentration device was inserted. The device was fabricated using two types of 3D printers: fused deposition modeling (FDM) (3 in 1 3D Printer, Snapmaker Co., Ltd., China) with a 1.75-mm diameter polylactic acid filament (Snapmaker Co., Ltd., China), and stereolithography apparatus (SLA) (Elegoo MARS, Elegoo Co., Ltd., China) with acrylic-based photopolymer resin (EKIMATE, NSS Co., Ltd., Japan). These printers featured different channel diameters of 1, 2, and 4 mm, each with a depth of 10 mm and flow channel volumes of 7.9 μL (Φ1 mm), 31.4 μL (Φ2 mm), and 125.7 μL (Φ4 mm), respectively.

To assess the effects of cyclone flow on the channel, we prepared variations with different channel positions and measured the liquid volume in the channel at the end of the concentration process. Channels with diameters of 1, 2, and 4 mm were positioned from the center to the edge of the device (d = 0 to 5 mm), as shown in Fig. 2b. The diameter determined the fluidic resistance of the cyclone, while the distance from the center influenced the coupling efficiency of the cyclones in the microtube and channel.

### Evaluation of recovery percentage after evaporation

A 32-mM dye solution (Sunset Yellow, Wako Pure Chemical Industries, Ltd. Japan) served as the standard sample for measuring the recovery rate. Two factors influenced the recovery percentage. First, when the applied pressure was low, the cyclone speed increased, generating droplets within the cyclone that were sucked into the vacuum pump, resulting in the loss of analyte molecules. Second, the adsorption of dye molecules onto the tube or channel surface contributed to the recovery percentage.

To quantify the concentration of the small volume samples after concentration, which was challenging to determine using a conventional light absorptiometer, a thermal lens detector (TLD) employing a coaxial laser (SDL-660-RG-100 T, Shanghai Dream Lasers Technology Co., Ltd., China) was utilized. The TLD enables the detection of small-volume light absorption by focusing the lasers and detecting heat following light absorption, allowing absorbance detection at less than 10^−3^ [23]. A 50-mW excitation laser with wavelengths of 532 nm for excitation and 660 nm for probing the thermal lens (TL) effect coaxially aligned. These lasers were focused through a lens (f = 10 mm, BK7, with a focal length difference of 10 mm for enhancing the TL signal) into a quartz cell with an optical path length of 1 mm (S10-SQ-1, GL Sciences Inc., Japan), creating a TLD. The excitation laser that passed through the quartz cell was filtered, and only the probe beam was introduced to a photodiode (PD) (ET-2030, Electro-Optics Technology, Inc., USA). To measure small signals from the PD, the excitation light was modulated at 630 Hz, and the synchronous signal intensity of the probe beam was detected using a lock-in amplifier (LI5640, NF Corporation Co., Ltd., Japan).

### Application to molybdenum ion detection in a river

As an application of the concentration device, the concentrations of molybdenum solutions were measured using a TLD with the Pack Test WAK-Mo (Kyoritsu Chemical-Check Lab., Co., Japan) and a 1000-ppm molybdenum standard solution (Wako Pure Chemical Industries, Ltd., Japan). Considering the 555-nm absorbance by the colorimetric reaction system of the equipment, the wavelength of the excitation laser was selected as 532 nm.

Molybdenum, a rare metal, is applied in mining, semiconductor substrates, and automobile parts due to its high melting point, hardness, and corrosion resistance. However, its high endurance leads to molybdenum being discharged into the environment. Screening molybdenum levels in environmental samples without expensive analytical instruments is crucial. Conventional equipment, however, has a detection sensitivity in the order of parts per million, which poses a problem of low sensitivity. Therefore, a method to detect molybdenum with high sensitivity in a simplified manner is required.

The lower limit of detection of the equipment was 5 ppm, which was deficient in detection sensitivity compared to the guideline value in Japan (70 ppb). Hence, a concentration device was required for environmental analysis. After establishing the calibration curve, a 1 mL diluted standard solution sample was concentrated 10 times (100 µL) and 33 times (30 µL). Moreover, the solution was compared with the theoretical values. Various contamination effects need to be considered in actual environmental sample measurements. To validate the application to environmental samples, we prepared a 50-ppb sample solution by diluting 0.5 µL of molybdenum standard solution with environmental water samples collected from rivers in Kanagawa and Fukuoka Prefecture, which brought the total volume to 10 mL.

## Results and discussion

**Fig. 3:**
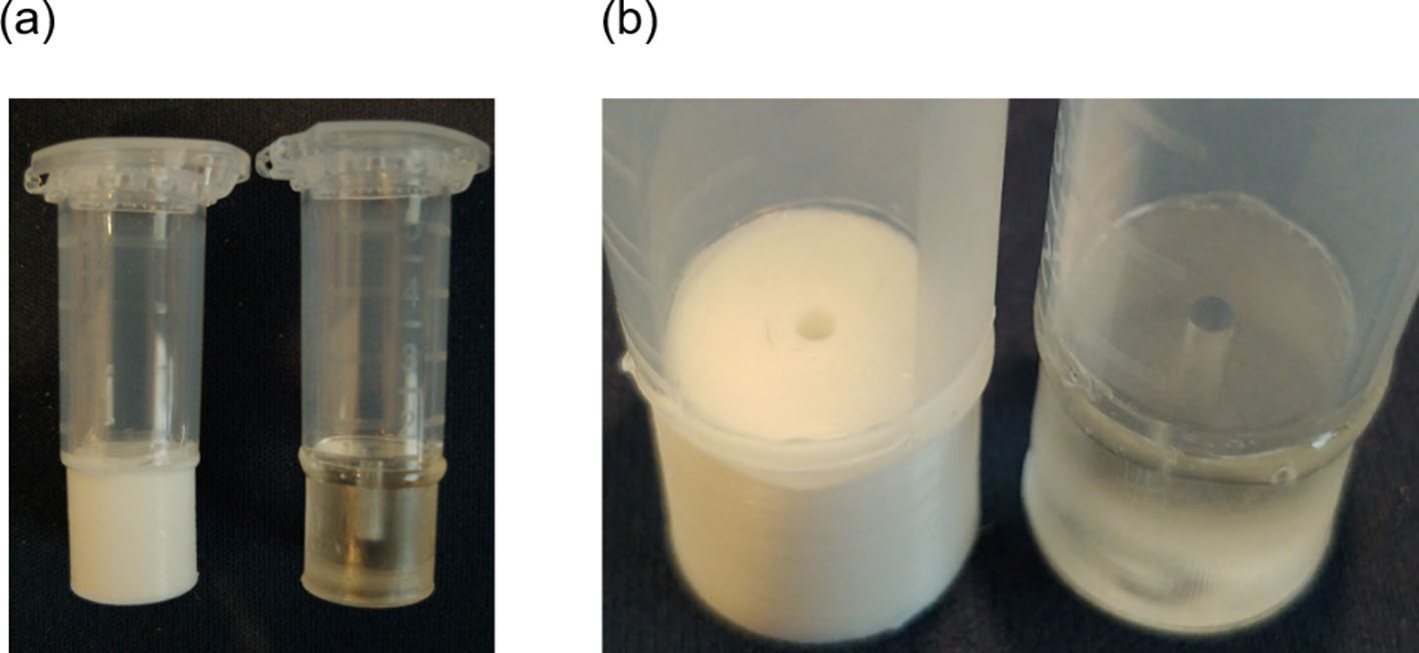
Cyclone quantitative concentration device fabricated using a 3D printer

The fabricated device is shown in Fig. 3. In the device fabricated by FDM, bubble formation inside the channel was observed, which presented difficulties in using the concentration device. This may be attributed to the formation of small air gaps in the polymer material due to low spatial resolution, introducing bubbles into the channel when applying negative pressure inside the microtube. Conversely, bubble formation was not observed in the case of SLA, and the concentration device worked well. Therefore, the device was fabricated using the SLA method. For the insert, the microtube was cut, and the quantitation unit with a millimeter-scale channel was bonded to the microtube.

**Fig. 4:**
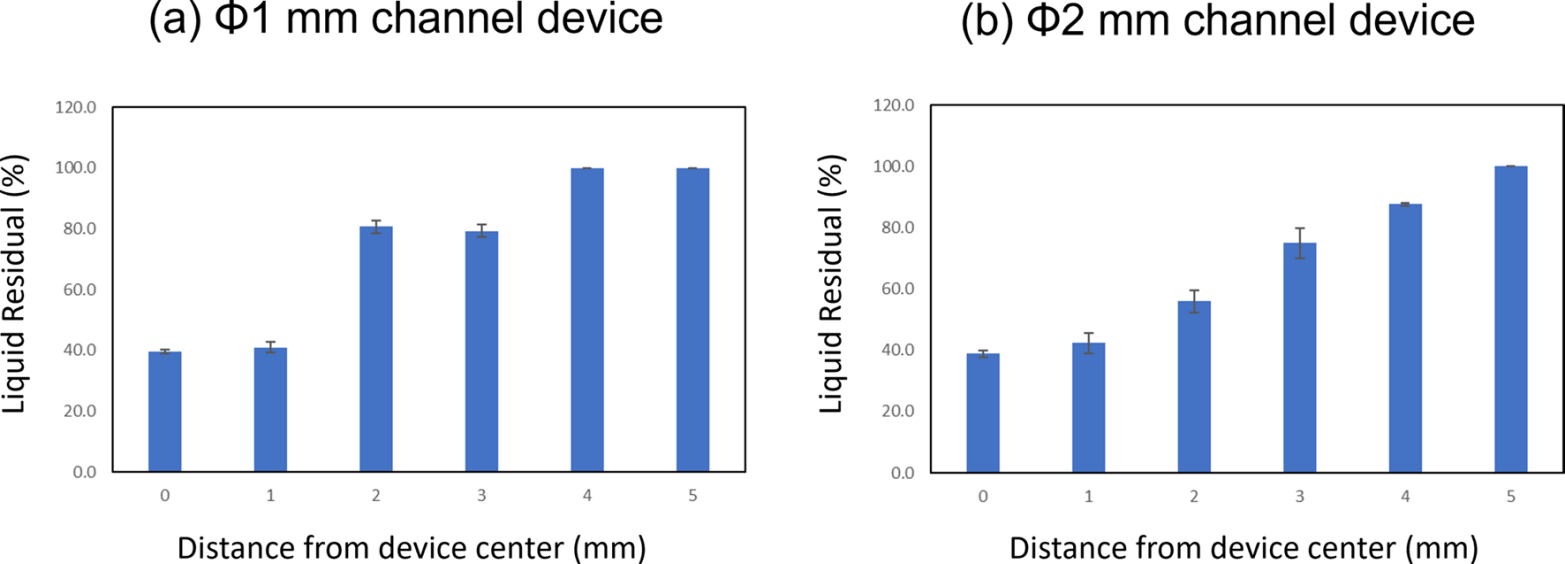
Results of the recovery rate of the liquid sample with the position of the channel for quantitative concentration

A comparison of the liquid residual for each channel size and position is shown in Fig. 4 and Table 1. A 1-mL sample of distilled water was concentrated at a rate of 30 μL/min, with concentration completed in 30 min. The quantity of liquid residual in the channel was measured as a percentage of the channel volume.

**Table 1:** Comparison of channel size and position

No	Diameter (mm)	d (mm)	Drying rate in channel (μL/min)	Cyclone State in channel
1	1	0	1.6	✓
2		1		✓
3		2	0.8	✓
4		3		✓
5		4		
6		5		
7	2	0	6.3	✓
8		1		✓
9		2		✓
10		3	3.1	✓
11		4		✓
12		5		
13	4	0	18.8	✓
14		1		✓
15		2		✓
16		3		✓
17		4		✓

For a Φ 1 mm channel, at d = 1 mm, 40% of the channel volume sample remained; at d = 2 and 3 mm, 80% of the channel volume sample remained; and at d = 4 and 5 mm, the sample remained at 100% of the channel volume. In the case of Φ 2 mm: at d = 1 mm, approximately 40% of the channel volume sample remained in the channel; at d = 2 to 4 mm, the sample volume increased; and at d = 5 mm, 100% of the channel volume liquid remained. Finally, in the Φ4 mm channel, the sample was dried entirely at all positions.

If the channel is either centrally located or large in size, the liquid starts concentrating inside the channel due to the cyclonic airflow before it has completely concentrated outside the channel in the container. Under optimized conditions, the liquid should fully concentrate in the container, and the process should be stopped before the liquid begins to concentrate in the channel. However, this simultaneous concentration increases the complexity of storing the liquid in the channel. Moreover, in cases where the liquid residual was below 80%, the sample formed a cyclone with a height of 1 to 2 mm in the channel. The liquid drying time was fast in the cyclone flow. Samples in each channel created in the center of the device lost 10–20% of their volume within 1 min. To store samples in the channel, cyclone flow in the channel was stopped to reduce the drying rate.

These results indicate that samples were stored in the channels fabricated with Φ1 mm and d = 4 or 5 mm, as well as Φ2 mm and d = 5 mm. Subsequently, we assessed the solute concentration of the sample under these conditions.

For verification of the liquid concentration level, a sunset yellow solution was used, and sample concentrations were measured after quantitative concentration using devices with Φ1-mm and Φ2-mm channels fabricated 5 mm outside from the center of the device, along with a 532-nm excitation TLD. The calibration curve using diluted standard solutions was almost linearly related in the range of 640 nM to 32 mM, with an R^2^ value exceeding 0.99. Subsequently, the standard solution sample (640 nM) was concentrated 10 times by each concentration device, and the concentrations were determined by TLD. Results revealed a 39% recovery rate of the theoretical value for the Φ1-mm channel device. In contrast, the Φ2-mm channel device showed a 96% recovery rate (Table 2). In the case of the Φ2-mm channel, the cyclone flow of the sample extended to the channel, allowing the proper mixing and concentration. Conversely, in the Φ1-mm case, the flow did not reach the whole channel, resulting in inadequate mixing and subsequent drying and adsorption of most of the sample onto the tube wall. Based on these results, the Φ2-mm channel device positioned at d = 5 mm was selected for quantitative concentration.

**Table 2:** Comparison of the recovery rate in the channel

Channel Diameter	Theoretical concentration (μM)	Theoretical TLD signal (μV)	TLD Signal (μV)	Recovery rate (%)	RSD (%)
1 mm	32	894.5	350.0	39.1	1.5
2 mm	32	894.5	860.0	96.1	2.1

Furthermore, molybdenum standard solutions were measured using a screening kit and TLD. The calibration curve using diluted standard solutions showed almost a linear relationship in the range of 250 ppb to 10 ppm, with an R^2^ value exceeding 0.99. Subsequently, the standard solution sample was concentrated 10 and 33 times by the concentration device, and the concentrations were determined by TLD. Recovery rates of 93% and 95% of the theoretical enrichment value were obtained (Table 3a). Table. 3b shows the results of using real samples from environmental water, where a molybdenum standard solution was added to river water to set the concentration below the environmental standard (final conc: 50 ppb) and concentrated 10 times. Compared to the theoretical value from the calibration curve, 85.4 and 83.3% recovery rates were confirmed, allowing the sample to be measured. The lower recovery rate may be attributed to the aspiration of small droplets created by the cyclone and adsorption to the container. In experiments using sunset yellow and molybdenum standard solutions diluted with distilled water, the recovery rates were 90% or higher. Therefore, this result suggests the possibility of adsorption onto the container, depending on the environmental sample. Environmental samples contain many contaminants, and molybdenum might adsorb and bind to these contaminants. Moreover, the material of the 3D printer device was adhesive, and the surface roughness was several micrometers due to the 25-µm layer construction. Hence, the adsorption of molybdenum and contaminants onto the 3D printer unit could have reduced the recovery rate. To solve this problem, we are considering fabricating devices by injection molding using low-adhesion materials. Injection molding enables the creation of a nanometer-level surface roughness by polishing the molds. Additionally, surface treatment and coatings are effective solutions that will be explored in the future.

**Table 3:** Results of the quantitation of molybdenum

a Concentration using a pack test				
Concentrated volume	Theoretical TLD signal (μV)	TLD Signal (μV)	Recovery rate (%)	RSD (%)
100 μL (× 10)	161.0	150.7	93.6	5.8
30 μL (× 33)	384.7	367.8	95.6	2.4

b In environmental water using concentration				
Region	Theoretical TLD signal (μV)	TLD Signal (μV)	Recovery rate (%)	RSD (%)
Kanagawa	48.0	41.0	85.4	4.9
Fukuoka	48.0	40.0	83.3	2.5

## Conclusion

In this study, we developed a device to quantitatively concentrate liquid samples by integrating the cyclone concentration method with a fluidic device, enhancing the detection sensitivity of the analysis for general use. The sizing and positioning of the device’s solution quantitative channel were determined, while the low-volume quantitative detection method using a TLD enabled the measurement of molybdenum in the order of ppb in environmental samples.

We plan to develop this device to include a sample dispensing function, making it suitable for use as a pipette for sampling. Additionally, we aim to extend its application to the measurement of other samples, such as biological substances.

## Supplementary Information

Below is the link to the electronic supplementary material.

Supplementary file1 (DOCX 621 KB)

## Data Availability

The source data for all figures are available upon request from the corresponding author K. Mawatari.
