# Supplementary material for: Room-temperature mL-to-μL quantitative liquid concentration device for cyclone flow
Source: Anal Sci. 2024 Aug 30;40(12):2175–80. doi: 10.1007/s44211-024-00654-z (PMC11588970; doi:10.1007/s44211-024-00654-z)
Supplement: Supplementary file 1 — Supplementary file1 (DOCX 621 KB) [file 44211_2024_654_MOESM1_ESM.docx]

**Supplementary Information**

**Room-temperature mL-to-μL quantitative liquid concentration device for cyclone flow**

**Analytical Sciences**

Hidekatsu Tazawa* and Kazuma Mawatari*

Graduate School of Information, Production and Systems, Waseda University

2-7 Hibikino, Wakamatsu, Kitakyushu city, Fukuoka, 808-0135, Japan.

*Corresponding Author.

E-mail: kmawatari@waseda.jp

tazawa@aoni.waseda.jp

**Methods of low volume quantitation.**

**
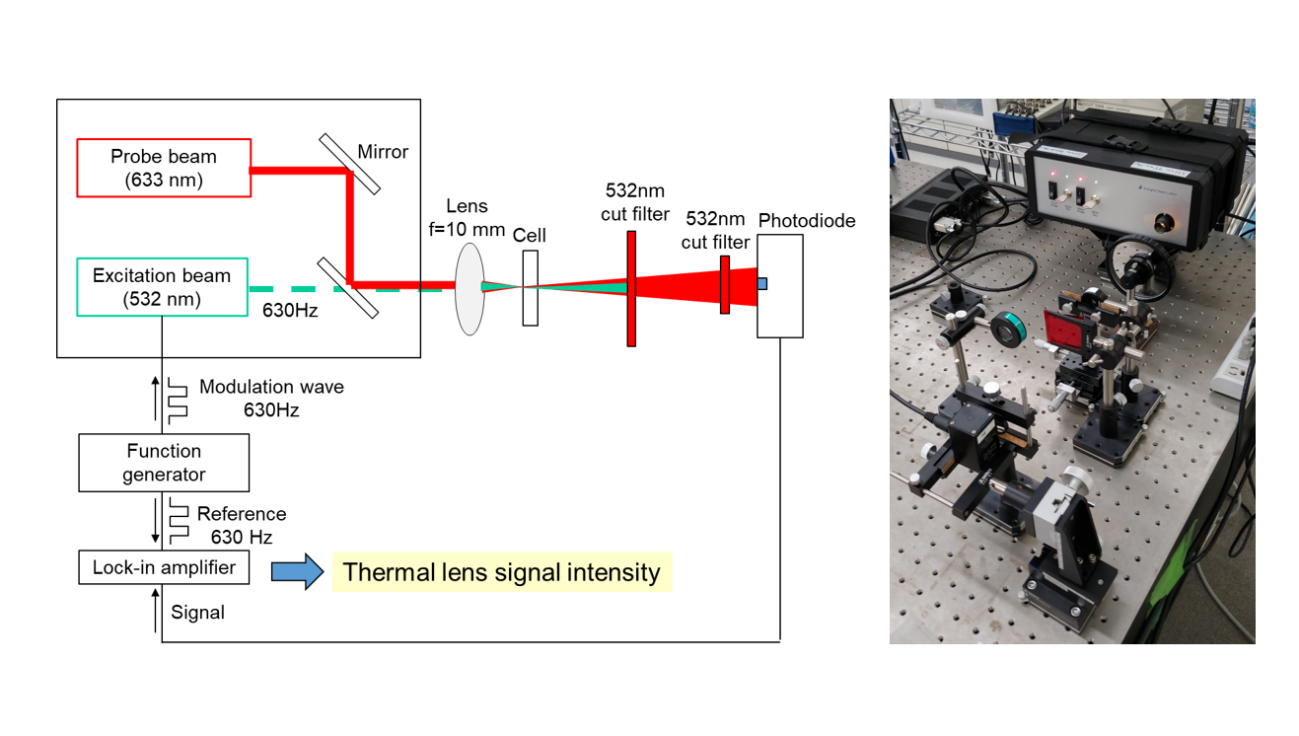
**

**Fig. S1** Optical design of thermal lens detection system
